# A mechanism-based pharmacokinetic/pharmacodynamic analysis of polymyxin B-based combination therapy against carbapenem-resistant *Klebsiella pneumoniae* isolates with diverse phenotypic and genotypic resistance mechanisms

**DOI:** 10.1128/aac.00782-25

**Published:** 2025-12-19

**Authors:** Ramya Mahadevan, Estefany Garcia, Rajnikant Sharma, Hongqiang Qiu, Ahmed Elsheikh, Robert Parambi, Cely Saad Abboud, Fernando Pasteran, Maria Soledad Ramirez, Keith S. Kaye, Robert A. Bonomo, Gauri G. Rao

**Affiliations:** 1 Titus Family Department of Clinical Practice, USC Alfred E. Mann School of Pharmacy and Pharmaceutical Sciences, University of Southern California 5116, Los Angeles, California, USA; 2 Division of Pharmaceutics and Experimental Therapeutics, Eshelman School of Pharmacy, University of North Carolina at Chapel Hill 15521, Chapel Hill, North Carolina, USA; 3 Department of Pharmacy, Fujian Medical University Union Hospital 117890, Fuzhou, People’s Republic of China; 4 College of Pharmacy, Fujian Medical University 74551, Fuzhou, People's Republic of China; 5 Instituto Dante Pazzanese de Cardiologia 67771, São Paulo, Brazil; 6 Antimicrobianos, Instituto Nacional de Enfermedades Infecciosas, Antimicrobial Service of the National Institute of Infectious Diseases (ANLIS Dr. Carlos G. Malbrán) 668363 , Buenos Aires, Argentina; 7 Center for Applied Biotechnology Studies, Department of Biological Science, College of Natural Sciences and Mathematics, California State University Fullerton 118567, Fullerton, California, USA; 8 Division of Allergy, Immunology and Infectious Diseases, Rutgers Robert Wood Johnson Medical School 12287 , New Brunswick, New Jersey, USA; 9 Department of Molecular Biology and Microbiology, Case Western Reserve University School of Medicine 12304, Cleveland, Ohio, USA; 10 CWRU-Cleveland VAMC Center for Antimicrobial Resistance and Epidemiology (Case VA CARES) 2546, Cleveland, Ohio, USA; 11 Geriatric Research Education and Clinical Center, Louis Stokes Cleveland Department of Veterans Affairs Medical Center, Education and Clinical Center 273136, Cleveland, Ohio, USA; 12 Department of Medicine, Case Western Reserve University School of Medicine 12304, Cleveland, Ohio, USA; 13 Departments of Pharmacology, Biochemistry, Proteomics and Bioinformatics, Case Western Reserve University School of Medicine 12304, Cleveland, Ohio, USA; Providence Portland Medical Center, Portland, Oregon, USA

**Keywords:** carbapenem resistant* Klebsiella pneumoniae*, mechanism based PK/PD model, polymyxin B based combination therapy

## Abstract

Increased resistance to β-lactams/β-lactamase inhibitors by mutations in β-lactamase genes, porins, and efflux pumps complicates the management of carbapenem-resistant *Klebsiella pneumoniae* (CRKP). Polymyxin B (PMB)-based combination therapy is the best alternative treatment for middle and low-income countries that cannot access the latest medicines. It is crucial to know both phenotypic and genotypic characteristics of a pathogen to understand the killing effect of each drug and its combinations. Hence, our objective was to incorporate mechanistic insights gained from resistance mechanisms of each isolate to develop a mechanism-based pharmacokinetic/pharmacodynamic model. Six clinical CRKP isolates with diverse genotypic resistance expressing *bla*_KPC_, *bla*_NDM_, porin, and mgrB mutations were used for static concentration time kill (SCTK) assays to evaluate the rate and extent of killing by monotherapy, double and triple combinations using PMB (0.5–64 mg/L), meropenem (10–120 mg/L), and fosfomycin (75–500 mg/L). Isolate BRKP28 expressed non-functional MgrB (a regulatory protein) and high-level phenotypic resistance (PMB MIC: >128 mg/L). In line with the observed resistance, the model estimated that BRKP28 had a reduced maximum killing rate constant for PMB (3.61 h⁻¹) relative to other isolates. The mechanistic synergy of PMB, due to outer membrane disruption, was incorporated into three isolates with porin mutations. PMB demonstrated 83%–88% mechanistic synergy with meropenem and 81%–98% with fosfomycin. The model further estimated that a very low concentration of PMB (0.49–0.64 mg/L) was sufficient to achieve 50% of the maximum synergy. Simulations using population pharmacokinetic models showed that combination therapy of PMB (1 mg/kg q12h) and fosfomycin (8 g q8h) achieved >73% reduction in area under the bacterial load-versus-time curve across four isolates. The triple combination therapy achieved a 67.7% reduction in non-carbapenamase producing isolate. These findings demonstrates that a low PMB dosing regimen (1 mg/kg q12h) can produce synergistic effects in combination therapy and may be effective in managing infections caused by CRKP, including PMB resistant isolates.

## INTRODUCTION

Infections caused by carbapenem-resistant Enterobacterales (CRE) are a significant public health crisis, as the development of new antibiotics is not keeping pace with the rapid rise in antimicrobial resistance (AMR). The global incidence of CRE has doubled in just two years, from an average of 4.2% in 2018 to 8.04% between 2020 and 2022 (1). In the United States, 83% of clinical CRE isolates are carbapenemase-producing, with the most prevalent β-lactamase (*bla*) genotypes being *bla*_KPC_ (80%), followed by *bla*_NDM_ (15%), *bla*_OXA-48_ (7%), and *bla*_IMP_ (5%). Between 2019 and 2021, *bla*_KPC_ prevalence decreased 1.3-fold, while isolates carrying other resistance genes increased by 5- to 8-fold. Non-carbapenamase-producing CRE isolates typically exhibit resistance through other mechanisms, including extended-spectrum β-lactamases (ESBLs), disruptions in outer membrane porin, and/or overexpression of genes encoding efflux pumps (2).

A comprehensive meta-analysis has demonstrated that integrating rapid molecular diagnostics (RMDs) to identify pathogens responsible for bloodstream infections with antimicrobial stewardship programs significantly lowers mortality rates, reduces the time to effective therapy, and shortens hospital stays (3). Recent studies, PRIMERS I and II (Platforms for Rapid Identification of MDR-Gram negative bacteria and Evaluation of Resistance Studies), have evaluated the accuracy of RMD platforms in identifying *bla* genotypes, which confer β-lactam resistance, to assist in the appropriate selection of β-lactams (4). Collectively, these studies underscore the importance of RMDs in the global effort to combat AMR.

The recent approval of novel β-lactamase inhibitors including avibactam, relebactam, vaborbactam, taniborbactam, and enmetazobactam has significantly advanced efforts to reduce CRE-related infections (5, 6). Additional inhibitors including ledaborbactam, zidebactam, xeruborbactam, funobactam, and nacubactam are in late-stage development or nearing approval (7). However, resistance to β-lactam/β-lactamase inhibitors has already been observed (8). Key resistance mechanisms include mutations in *bla*_KPC_, changes in outer membrane permeability, and the presence of *bla*_NDM_ (2).

The diversity of resistant mechanisms in CRE makes it challenging to identify appropriate therapies that are effective in managing CRE infections. In the absence of new antimicrobial agents, optimizing the use of existing antibiotics is a crucial strategy to combat AMR (9). A multi-study analysis of patients infected with carbapenem-resistant *Klebsiella pneumoniae* (CRKP) found that combination therapy reduced mortality rates to 25%–31%, a 2- to 2.35-fold improvement over monotherapy with mortality rates of 50%–73% (10–12). Current polymyxin guidelines recommend polymyxin B-based combinations as an effective alternative for CRE infections, though their nephrotoxicity limits widespread use (13). Recent clinical trials, including OVERCOME and AIDA, have shown reduced mortality with colistin-meropenem combination therapy compared to monotherapy (14, 15).

Despite recent advancements, clinicians continue to face challenges in selecting the most appropriate antibiotic combinations for CRE infections. Fosfomycin, a bactericidal drug with a favorable safety profile and a distinct mechanism of action (inhibiting the peptidoglycan synthesis at an earlier stage) than β-lactams, has emerged as a promising candidate for combination therapy against Gram negative pathogens. Notably, fosfomycin has demonstrated *in vitro* and *in vivo* synergy with both meropenem and polymyxin B, and this synergy appears unaffected by polymyxin resistance-associated mutations (*mgrB*, *crrB*, *pmrA*, *pmrB*, *pmrC*) or the presence of β-lactamases (16).

Given that an antibiotic’s spectrum of activity is shaped by the underlying resistance mechanism (carbapenemase producing CRKP vs non-carbapenamase producing CRKP), a systematic and rational approach that integrates resistance mechanisms and susceptibility data is necessary for effective management of CRE infections.

To this end, static concentration time-kill assays were performed to evaluate the impact of treatment with polymyxin B, meropenem, and fosfomycin as monotherapies, as well as polymyxin B-based combinations with either fosfomycin or meropenem, and a triple combination of all three drugs against six CRKP clinical isolates representing both carbapenamase producing and non-carbapenamase producing clinical isolates. The strains were genomically characterized to elucidate mechanistic insights into their killing activity based on the resistance mechanisms expressed. The objective of this study was to characterize the bacterial killing dynamics of six CRKP isolates by integrating their phenotypic and genotypic resistance profiles to inform the development of a mechanism-based pharmacokinetic/pharmacodynamic (PK/PD) model (MBM).

## RESULTS

### Susceptibility and resistance gene profiles for clinical isolates

The minimum inhibitory concentrations (MICs) and relevant resistance genes for the six isolates are summarized in Table 1. Among the six isolates, four isolates (BRKP61, BRKP67, BRKP76, BRKP28) expressed *bla*_KPC-2_ with outer membrane porin mutations. BRKP67 and BRKP28 also exhibited non-functional MgrB protein, conferring resistance to polymyxin B. The non-carbapenemase producer, KP0016-1 harbored outer membrane porin mutations, while KP0052-1, expresses *bla*_NDM_ without porin mutations. The complete gene characterization profiles for each isolate are provided in Table S2.

**TABLE 1:** Antimicrobial resistance genes and MICs for each of the six CRKP isolates^*a*^

	Resistance genes							
Isolate	Carbapenamase	*mgrB*	*ompK35*	*ompK36*	*ompK37*	PMB MIC (mg/L)	FOF MIC (mg/L)	MEM MIC (mg/L)
BRKP28	*bla*_KPC-2_	NF	NF	NF	Present	>128 R	128 R	256 R
BRKP61	*bla*_KPC-2_	F	NF	NF	Present	<0.5 I	256 R	128 R
BRKP67	*bla*_KPC-2_	NF	NF	NF	Present	8 R	32 R	64 R
BRKP76	*bla*_KPC-2_	F	NF	NF	NF	<0.5 I	32 R	64 R
KP0016-1	Not present	F	NF	NF	NF	<0.5 I	64 R	64 R
KP0052-1	*bla*_NDM-4_	F	Present	Present	Present	<0.5 I	64 R	64 R

^*a*^ NF, non-functional; F, functional, Polymyxin B and meropenem MICs were interpreted using CLSI breakpoints for *Klebsiella pneumoniae. *Fosfomycin MICs were interpreted using EUCAST breakpoint for *E. coli. *I, intermediate, R, resistant.

### Evaluation of pharmacodynamic effect with mono and combination therapy

**Fig 1:**
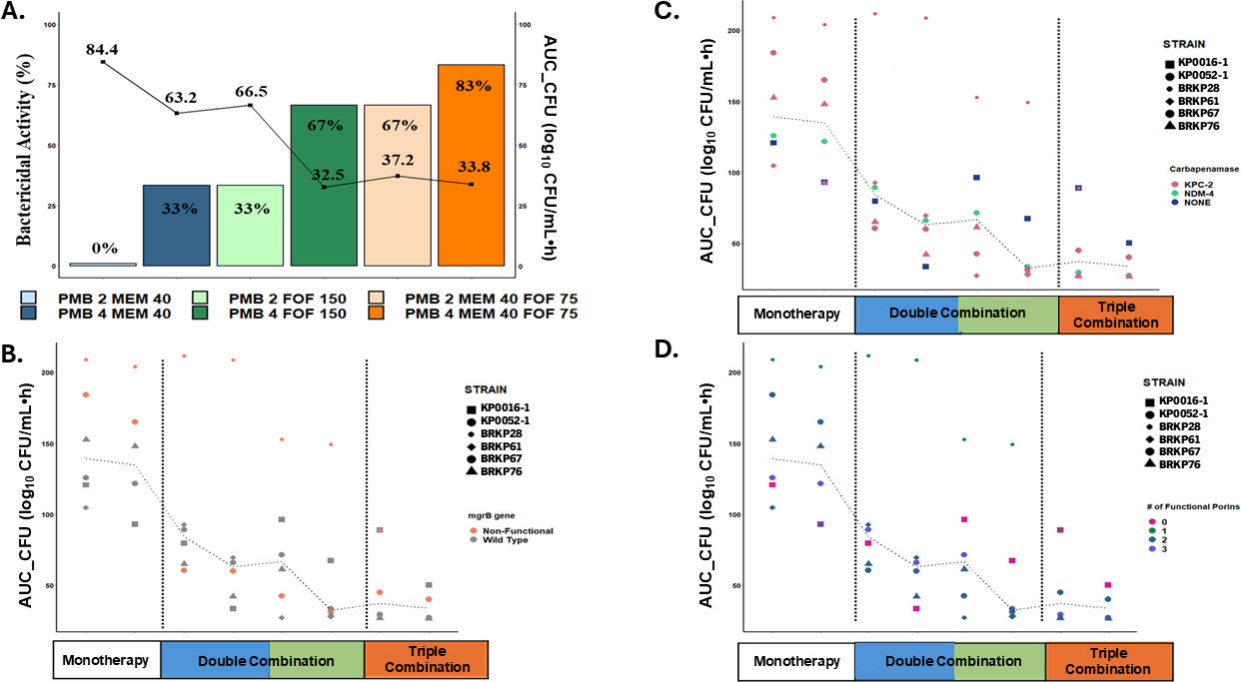
(**A**) illustrates the bactericidal activity and extent of bacterial reduction (AUC_CFU) for polymyxin B-based double and triple combinations. The bar graph represents the percentage of bactericidal activity, while the line graph shows the median AUC_CFU achieved by each drug regimen. (**B–D**) show the corresponding AUC_CFU for each of the six CRKP isolates, categorized by their respective resistance mechanisms: *mgrB* protein (**B**), carbapenemase production (**C**) and the number of functional porins (**D**).

Figure 1A summarizes the percentage of bactericidal activity and extent of reduction in bacterial burden over time (i.e., the area under the bacterial load-versus-time curve, AUC_CFU) achieved with each polymyxin B-based double and triple combinations. Bactericidal activity increased progressively from 33% with polymyxin B-meropenem, to 67% with polymyxin B-fosfomycin, and 83% with the triple combination. Increasing polymyxin B concentration from 2 to 4 mg/L enhanced bactericidal activity across all strains, in combination with meropenem (PMB-MEM: 0% to 33%), fosfomycin (PMB-FOF: 33% to 67%) and the triple combination (PMB-MEM-FOF: 67% to 83%). Overall, the triple combination showed greater bacterial reduction than double combinations, as reflected by lower AUC_CFU value of 33.8 log_10_ CFU·h/mL. The bacterial reduction was comparable between the higher concentration double combination (PMB 4 mg/L + FOF 150 mg/L) and the lower concentration triple combination (PMB 2 mg/L + MEM 40 mg/L + FOF 75 mg/L), with similar median AUC_CFU (range: 32.5–37.2 log_10_ CFU·h/mL).

Polymyxin B-based combinations were effective against isolates expressing carbapenemases and porin mutations (Fig. 1C and D). Enhanced pharmacodynamic activity was observed with polymyxin B combination therapy against KP0016-1 despite the absence of functional porins. In polymyxin B-resistant isolates, double combinations resulted in substantial bacterial reduction against BRKP67 but minimal activity against BRKP28 (Fig. 1B). The triple combination with higher polymyxin B concentrations (PMB 4 mg/L + MEM 40 mg/L + FOF 75 mg/L), yielded comparable bacterial reduction for both BRKP67 and BRKP28 (40.1 vs 50.3 log_10_ CFU·h/mL).

### Mechanism-based PK/PD model

**Fig 2:**
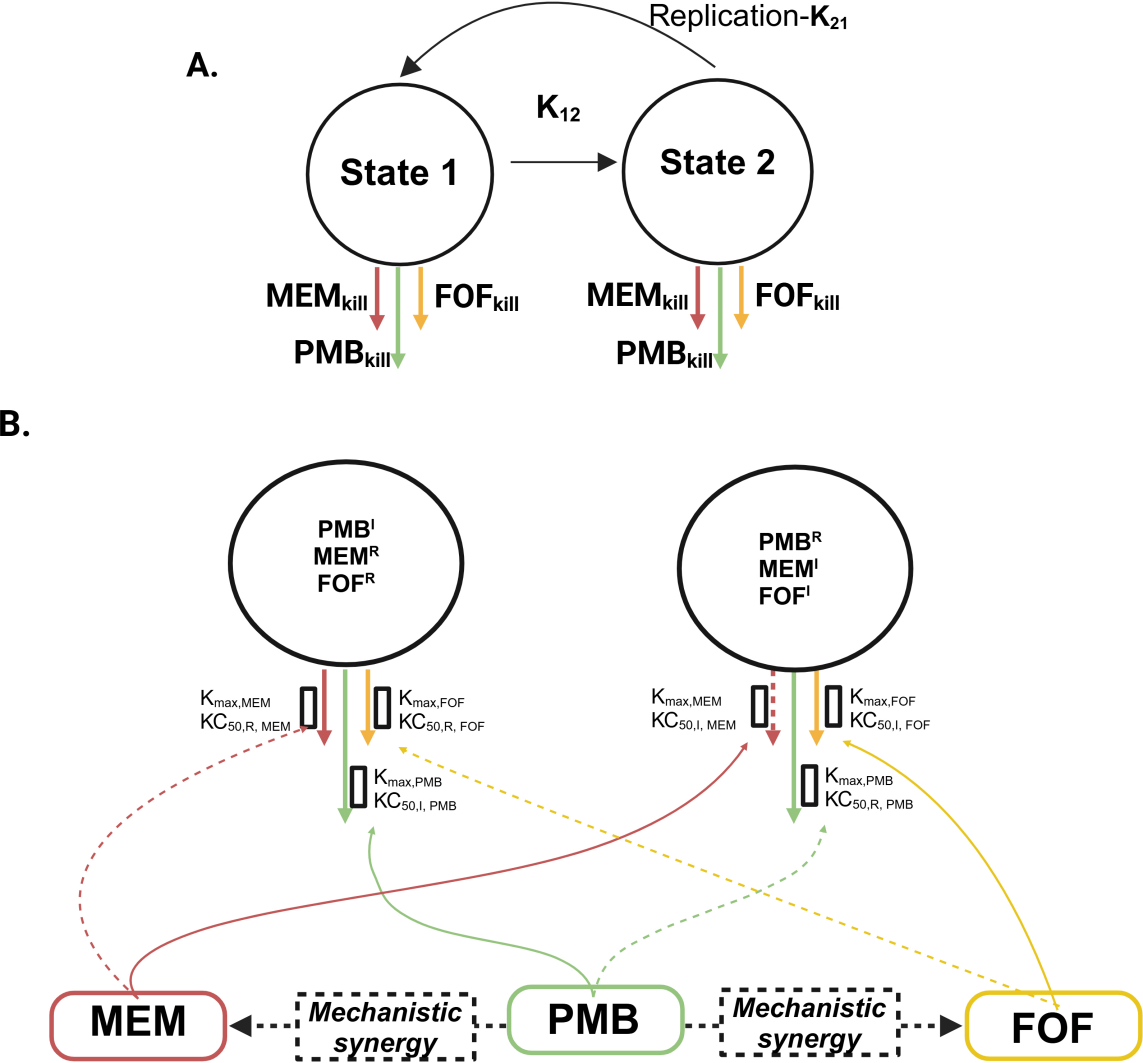
The mechanism-based PK/PD model structure describing the bacterial killing by meropenem (MEM_kill_), polymyxin B (PMB_kill_), and fosfomycin (FOF_kill_) as monotherapy and in combination therapy. (**A**) A two-state bacterial life cycle model was utilized to describe the bacterial replication and drug effect of bacterial killing is on both state 1 and state 2 of life cycle model. (**B**) The first subpopulation PMB^I^/MEM^R^/FOF^R^ is intermediately resistant to polymyxin B (PMB) and resistant to meropenem (MEM) and fosfomycin (FOF). The second subpopulation PMB^R^/MEM^I^/FOF^I^ is resistant to polymyxin B (PMB) and intermediately resistant to meropenem (MEM) and fosfomycin (FOF). The maximum killing rate constants (*K*_max_) and the associated concentrations causing 50% of *K*_max_ (*KC*_*50*_) are explained in Table 2. The structure also depicts the shift in meropenem (MEM) and fosfomycin (FOF) *KC*_*50*_ resulting from the mechanistic synergy because of polymyxin B (PMB) impacting MEM/FOF – intermediate/resistant subpopulation.

The mechanism-based PK/PD model structure shown in Fig. 2 describes the time-kill dynamics for six isolates, incorporating both subpopulation synergy (where polymyxin B targets bacterial populations resistant to meropenem and/or fosfomycin, and vice versa) and mechanistic synergy. Among the polymyxin B resistant isolates, BRKP28 (harboring a non-functional MgrB protein and polymyxin B MIC >128 mg/L) exhibited the lowest maximum killing rate constant for polymyxin B (*Kmax*_*PMB*_ = 3.61 h⁻¹) compared with other isolates (6.61–13.42 h⁻¹). Interestingly, BRKP67 which also carried an *mgrB* mutation but had a lower polymyxin B MIC (8 mg/L), showed a slightly higher *Kmax*_*PMB*_ of 9.08h⁻¹. Despite similar resistance mutations, the reduced killing effect of polymyxin B against BRKP28 may be due to additional uncharacterized resistance mechanisms.

The non-carbapenemase-producing isolate KP0016-1, carrying three porin mutations, showed the lowest killing rate constant for both meropenem (1.56 h⁻¹) and fosfomycin (0.64h⁻¹). Among isolates with a similar meropenem MICs of 64 mg/L, the *bla*_NDM-4_ producer displayed a lower *Kmax* for meropenem (3.05 h⁻¹) than *bla*_KPC-2_ producers (4.16–5.41h⁻¹). Furthermore, BRKP61 (MIC: 128 mg/L) and BRKP28 (MIC: 256 mg/L), both *bla*_KPC-2_ producers with higher meropenem MICs demonstrated correspondingly lower *Kmax* values for meropenem (2.13 h⁻¹ and 3.70h⁻¹, respectively).

BRKP61, KP0016-1, and BRKP76 isolates carried porin mutations, which are assumed to reduce the penetration of hydrophilic drug molecules such as meropenem and fosfomycin. The mechanistic synergy of polymyxin B, through outer membrane disruption, was expected to enhance the target site exposure of both drugs in these isolates. For meropenem, the mechanistic synergy with polymyxin B resulted in *Imax,_M,PMB_* values of 0.84, 0.83, and 0.88 for BRKP61, BRKP76, and KP0016-1, respectively, with IC50 values (polymyxin B concentration required to achieve 50% of *Imax,_M,PMB_*) ranging from 0.49 to 0.51 mg/L. For fosfomycin, the *Imax,_F,PMB_* values were 0.99, 0.81, and 0.89 for BRKP61, BRKP76, and KP0016-1, respectively, with IC50 values between 0.58 and 0.64 mg/L.

**Fig 3:**
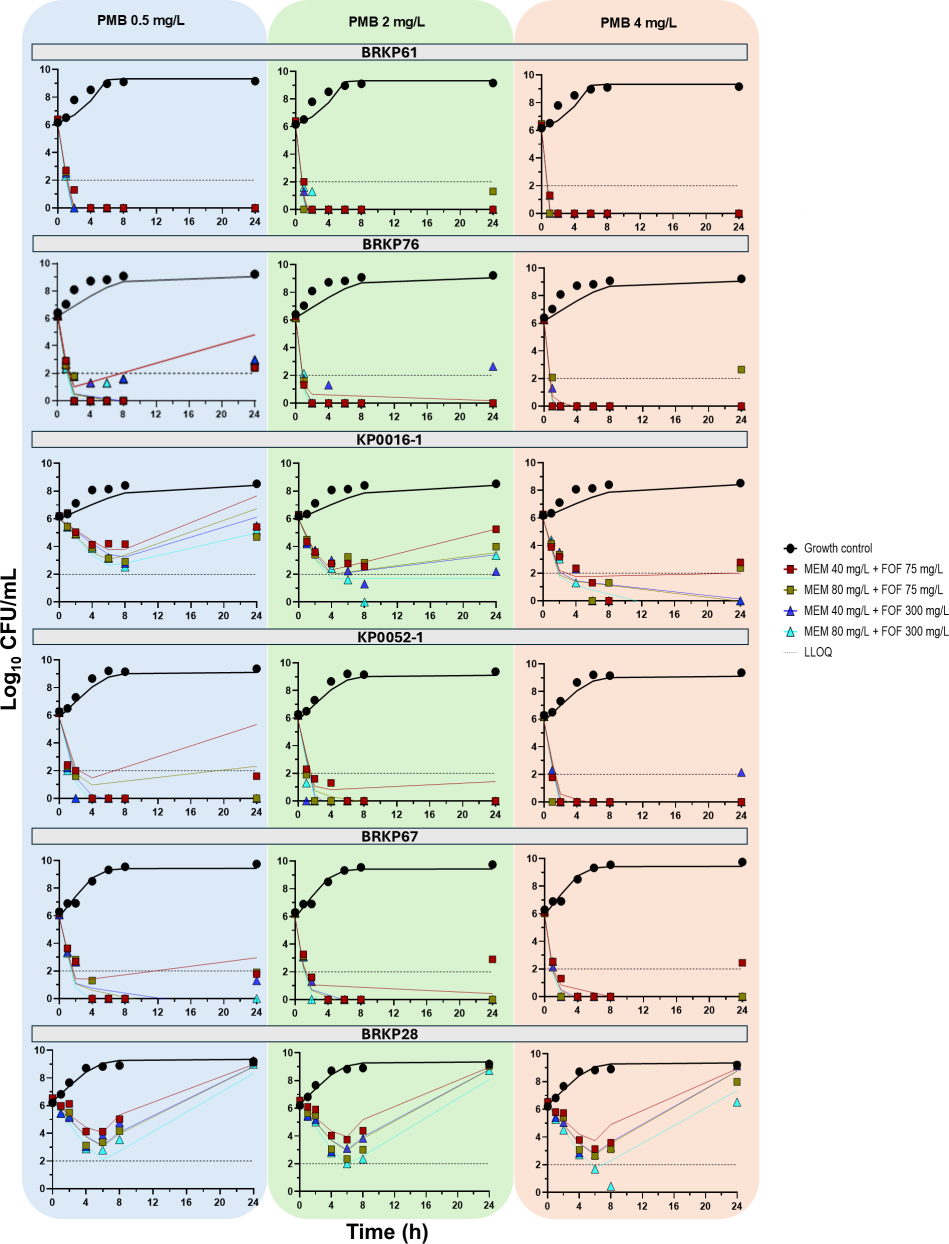
The model predictions for triple combination therapy with polymyxin B 0.5 mg/L, 2 mg/L, and 4 mg/L for six isolates are shown. The solid line represents the model predictions, while the symbols indicate the observed data. The horizontal dotted line marks the LLOQ (2 log_10_ CFU/mL).

In contrast, incorporating mechanistic synergy did not improve the model fits for isolates BRKP67 and BRKP28, both of which harbored MgrB and porin mutations. Based on resistance gene profiles and model discrimination, KP0052-1, which lacked porin mutations, was well described by subpopulation synergy alone. Correlation coefficients between observed and model predicted log_10_ CFU/mL values were greater than 0.73 across all isolates, indicating good model performance: BRKP61 (0.79), BRKP76 (0.73), KP0016-1 (0.84), KP0052-1 (0.74), BRKP67 (0.73), and BRKP28 (0.91) (Fig. S2). Parameter estimates for each isolate are provided in Table 2. Model predictions of bacterial load over time for triple combination (Fig. 3)**,** double combination (Fig. S3), and monotherapy (Fig. S4) aligned well with the observed data.

**TABLE 2:** Final model parameter estimates

Parameter estimate (%CV)	BRKP61	BRKP76	KP0016-1	KP0052-1	BRKP67	BRKP28
Bacterial growth and subpopulations						
Bacterial initial inoculum (LogCFU0) [log_10_ CFU/mL]	6.11 (0.64)	6.18 (2.20)	6.0 (1.04)	5.86 (5.18)	5.93 (1.64)	6.32 (0.45)
Maximum population size (LogCFUmax) [log_10_ CFU/mL]	9.33 (1.87)	9.05 (1.24)	8.43 (2.51)	9.10 (1.32)	9.44 (2.02)	9.35 (0.39)
Replication rate constant (k21) [h^−1^]	50 (fixed)	50 (fixed)	50 (fixed)	50 (fixed)	50 (fixed)	50 (fixed)
Mean generation time (MGT) [min]						
PMB^I^/MEM^R^/FOF^R^	83.3 (3.73)	–^*a*^	–	–	–	–
PMB^R^/MEM^I^/FOF^I^	13.5 (3.73)	–	–	–	–	–
PMB^I^/MEM^I^/FOF^I^	–	72.1 (7.71)	98.3 (5.07)	43.3 (8.07)	32.3 (12.5)	–
PMB^R^/MEM^R^/FOF^I^	–	–	–	–	–	44.25 (6.50)
PMB^R^/MEM^R^/FOF^R^	–	30.92 (4.74)	69.26 (2.31)	32.5 (5.0)	31.9 (5.08)	19.1 (7.39)
Log mutant frequency at baseline (log_10_ MF)						
PMB^R^/MEM^I^/FOF^I^	−5.10 (1.91)	–	–	–		–
PMB^R^/MEM^R^/FOF^R^		−5.48 (2.40)	−4.29 (3.14)	−5.15 (2.64)	−4.80 (1.64)	−6.55 (15.4)
Drug effect of polymyxin B						
Maximum killing rate constant of polymyxin B (Kmax_PMB_) [h^−1^]	13.4 (2.76)	11.2 (15.7)	10.9 (9.31)	6.61 (5.84)	9.08 (6.68)	3.61 (13.4)
Polymyxin B concentration causing 50% of Kmax_PMB_ in I (KC_50,PMB,I_) [mg/L]	0.82 (3.53)	2.20 (17.8)	6.56 (13.7)	2.68 (14.2)	2.77 (6.59)	–
Polymyxin B concentration causing 50% of Kmax_PMB_ in R (KC_50,PMB,R_) [mg/L]	41.03 (5.83)	79.2 (5.75)	61.16 (7.32)	38.34 (7.18)	96.78 (7.07)	38.82 (13.35)
Hill coefficient of polymyxin B	0.64 (3.83)	1.01 (8.25)	1.09 (4.14)	0.79 (5.62)	0.82 (10.16)	1.44 (20.82)
Drug effect of meropenem						
Maximum killing rate constant of meropenem (Kmax_MEM_) [h^−1^]	2.13 (4.39)	5.41 (5.04)	1.56 (12.9)	3.05 (18.8)	4.16 (7.09)	3.70 (16.5)
Meropenem concentration causing 50% of Kmax_MEM_ in I (KC_50,MEM,I_) [mg/L]	24.7 (6.50)	18.9 (12.4)	112 (9.39)	44.2 (16.5)	8.91 (25.8)	–
Meropenem concentration causing 50% of Kmax_MEM_ in R (KC_50,MEM,R_) [mg/L]	61.4 (4.30)	328 (2.50)	424 (2.83)	284 (11.5)	227 (12.3)	228 (11.1)
Hill coefficient of meropenem	1.85 (6.54)	1.26 (6.10)	1.99 (9.72)	0.84 (11.6)	1.17 (8.20)	1.43 (12.2)
Drug effect of fosfomycin						
Maximum killing rate constant of fosfomycin (Kmax_FOF_) [h^−1^]	3.74 (7.62)	3.03 (6.57)	0.64 (8.00)	4.01 (16.4)	3.45 (11.7)	3.42 (7.37)
Fosfomycin concentration causing 50% of Kmax_FOF_ in I (KC_50,FOF,I_) [mg/L]	42.5 (7.57)	21.1 (10.2)	20.2 (7.54)	26.1 (30.4)	20.4 (22.2)	22.0 (20.6)
Fosfomycin concentration causing 50% of Kmax_FOF_ in R (KC_50,FOF,R_) [mg/L]	44.71 (4.26)	647.5 (2.82)	448 (7.89)	761.7 (22.9)	1000 (7.38)	1342 (15.9)
Hill coefficient of fosfomycin	0.61 (9.59)	0.64 (27.7)	0.79 (22.5)	0.77 (8.81)	0.36 (9.64)	0.35 (23.73)
Mechanistic synergy of polymyxin B on Meropenem						
Maximum fractional decrease of KC_50,MEM,I_ and/or KC_50,MEM,R_ by polymyxin B via outer membrane disruption (Imax_M,PMB_)	0.84 (7.32)	0.83 (13.0)	0.88 (18.7)	–	–	–
Polymyxin B concentration causing 50% of Imax_M,PMB_ (IC50_M,PMB_) [mg/L]	0.51 (6.92)	0.49 (21.01)	0.50 (26.65)	–	–	–
Hill coefficient for the mechanistic synergy	0.88 (6.50)	0.54 (16.3)	0.63 (14.7)	–	–	–
Mechanistic synergy of polymyxin B on Fosfomycin						
Maximum fractional decrease of KC_50,FOF,I_ and/or KC_50,FOF,R_ by polymyxin B via outer membrane disruption (Imax_F,PMB_)	0.99 (9.89)	0.81 (14.5)	0.89 (13.6)	–	–	–
Polymyxin B concentration causing 50% of Imax_F,PMB_ (IC50_F,PMB_) [mg/L]	0.64 (9.35)	0.58 (34.8)	0.59 (15.7)	–	–	–
Hill coefficient for the mechanistic synergy	0.72 (7.22)	0.52 (14.4)	0.56 (12.3)	–	–	–
Variability model						
Additive residual variability [log_10_ CFU/mL]	0.39 (7.07)	0.54 (6.63)	0.45 (5.67)	0.77 (6.37)	0.52 (6.85)	0.25 (5.02)

^*a*^ –, parameter not part of the structural model for the corresponding strain.

### Model predicted bacterial load reduction in critically ill patients

**Fig 4:**
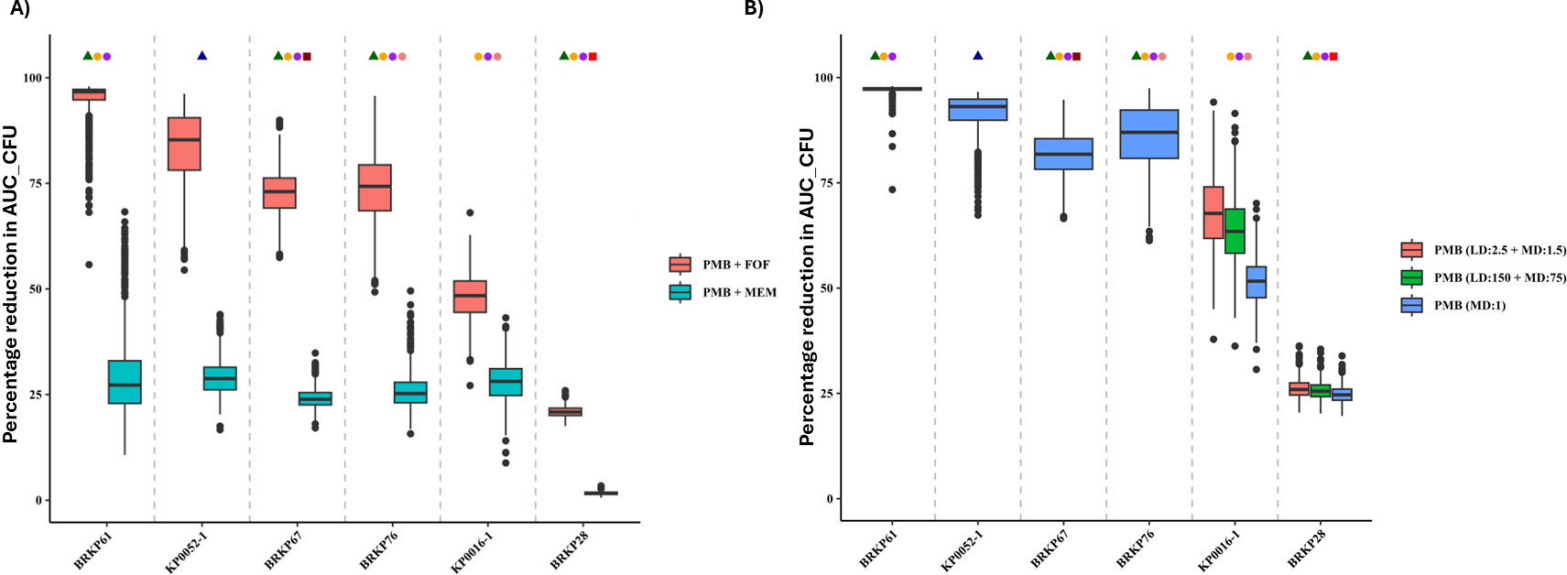
Box plots showing the percentage reduction in AUC_CFU for (**A**) double combination therapy with polymyxin B (1 mg/kg q12h) + meropenem (2 g q8h) or fosfomycin (8 g q8h) (**B**) Box plots showing the percentage reduction in AUC_CFU for triple combination therapy with different polymyxin B regimens. Fosfomycin was administered at 4 g q8h for BRKP61 and 8 g q8h for all other isolates. Meropenem was administered at 2 g q8h for two isolates KP0016-1 and BRKP28 and 1 g q8h for all other isolates. Green and blue triangle represents isolates with *bla*_KPC-2_ and *bla*_NDM-4_ respectively. Orange, purple, and brown circles indicate the presence of *ompK-35, ompK-36,* and *ompK-37* mutations, respectively. The brown square represents the insertion of ISKpn13 (1,148 bp), an IS5-like element in the *mgrB*, with a polymyxin B MIC of 8 mg/L. The red square represents a premature stop codon in the MgrB protein, with a polymyxin B MIC of > 128 mg/L.

Simulations using a population PK model for critically ill patients showed median unbound polymyxin B exposures of 39.7 mg·h/L and 49.0 mg·h/L for recommended regimens: LD 2 mg/kg + MD 1.25 mg/kg q12h and LD 2.5 mg/kg + MD 1.5 mg/kg q12h, respectively. A previously recommended fixed dosing regimen (LD 150 mg + MD 75 mg q12h) yielded unbound polymyxin B exposure of 39.9 mg·h/L, similar to the lower bound of the range observed with the recommended weight-based regimen (17). A lower dose polymyxin B regimen (1 mg/kg q12h) resulted in a median exposure of 23.5 mg·h/L, which was 41%–52% lower than the recommended regimens. Fig. 4 presents box plots of percentage reduction in AUC_CFU for six isolates treated with double and triple combination therapy, and the time course log_10_ CFU/mL simulation data are shown in Fig. S5.

The double combination of polymyxin B (1 mg/kg q12h) with meropenem (2 g q8h) achieved <30% reduction in AUC_CFU for all six isolates; however, all isolates except BRKP28 exhibited an initial ~2 log_10_ CFU/mL reduction within the first 4 h, followed by regrowth Fig. S5. For BRKP61 isolate (*bla*_KPC-2_ + two porin mutations), the combination of polymyxin B (1 mg/kg q12h) with fosfomycin (8 g q8h) reduced AUC_CFU by 96.7%. The triple drug combination achieved a similar reduction (97.3%) when a lower fosfomycin dose (4 g q8h) was combined with lower meropenem (1 g q8h) and polymyxin B (1 mg/kg q12h) doses. Similarly, KP0052-1 (*bla*_NDM-4_ with no porin mutations) showed 85.3% reduction with polymyxin B (1 mg/kg q12h) and fosfomycin (8 g q8h), while the triple combination with same polymyxin B, fosfomycin doses and a lower meropenem dose (1 g q8h) resulted in a 93.1% reduction in AUC_CFU.

The double combination of polymyxin B (1 mg/kg q12h) and fosfomycin (8 g q8h) resulted in 74.3% and 73.0% reduction in AUC_CFU for BRKP76 (*bla_KPC-2_* + three porin mutations) and BRKP67 (*bla_KPC-2_* + two porin + *mgrB* mutations), respectively. Adding meropenem (1 g q8h) in the triple combination further improved the percentage reduction in AUC_CFU by ~1.1-fold (BRKP76: 87.0% and BRKP67: 81.8%).

**Fig 5:**
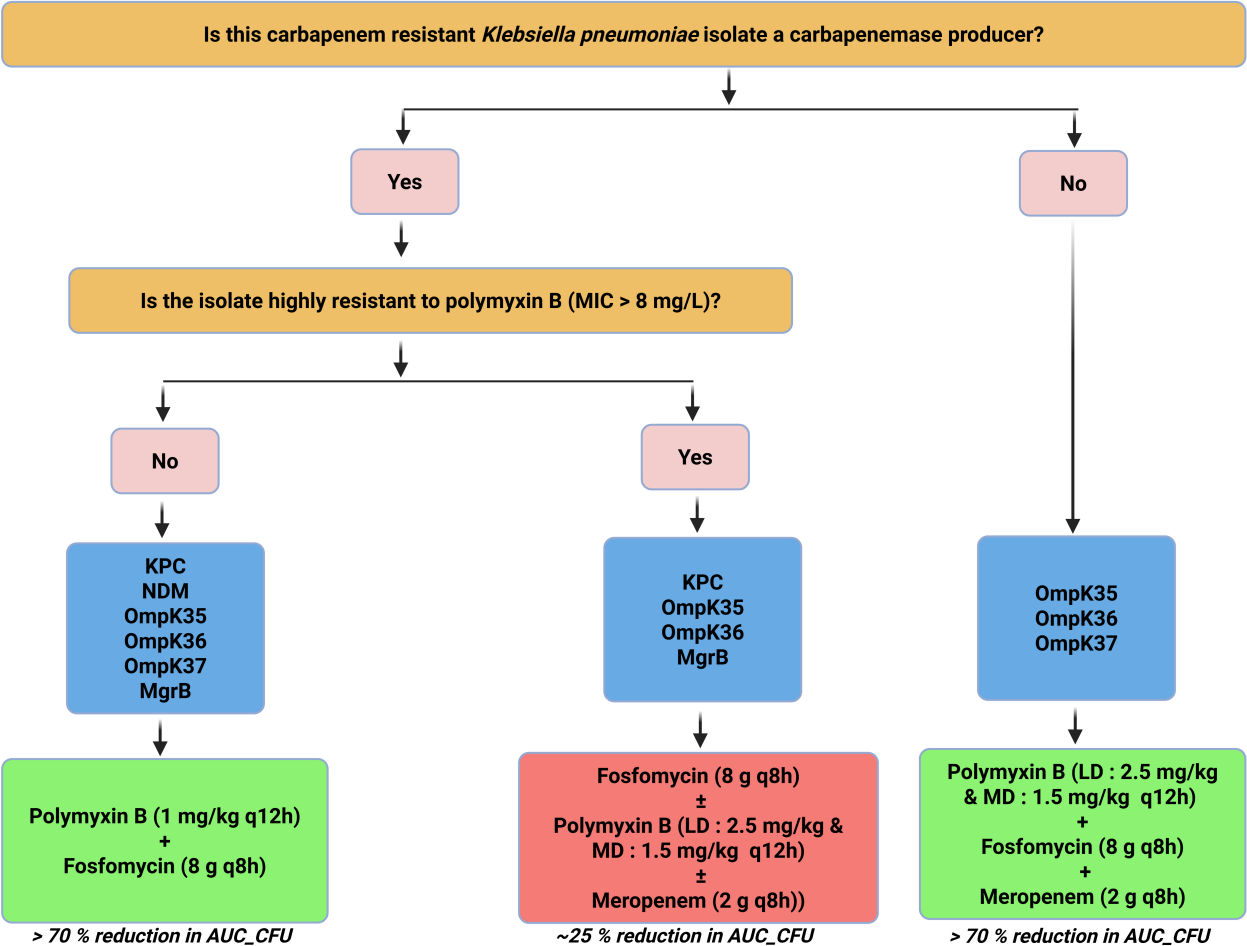
Workflow for selecting polymyxin B-based combination therapy. The decision pathway is based on carbapenemases production and polymyxin B susceptibility. Yellow boxes represent the critical questions used to determine the treatment regimens. Pink boxes represent decision points. Blue boxes show the different resistance genes present among the six isolates. Green boxes represent treatment regimens that were successful, achieving > 70% reduction in AUC_CFU. Red box indicates an unsuccessful treatment regimen, with only a 25% reduction in AUC_CFU.

For KP0016-1 (non-carbapenemase producer with three porin mutations), the triple drug combination with median unbound polymyxin B exposure of 23.5 mg·h/L resulted in only a 51.7% reduction in AUC_CFU. Increasing the median exposure to 49.0 mg·h/L enhanced the reduction to 67.7%. BRKP28, a strain resistant to all three tested antibiotics, exhibited only a modest reduction in bacterial load (20.9%) with fosfomycin alone or in combination with polymyxin B. The triple-drug regimen also failed to produce a meaningful reduction. Notably, fosfomycin alone led to a substantial initial decrease of 2–3 log_10_ CFU/mL within the first 8 h. Fig. 5 outlines the overall workflow for selecting polymyxin B-based combination therapy based on carbapenamase production and polymyxin B susceptibility.

## DISCUSSION

The rising incidence of both carbapenemase producing and non-carbapenemase producing CRKP infections, coupled with a lack of effective treatment options, underscores the need for detailed *in vitro* evaluation of potential treatment regimens using currently approved drugs. Although newer β-lactams/β-lactamase inhibitor combinations are recommended for treating carbapenemase producing CRKP, there is increasing evidence of resistance due to overexpressed efflux pumps, porin loss, or mutations in carbapenemases (18–21). Several studies have investigated the efficacy of polymyxin B-based combinations against NDM-producing *K. pneumoniae,* reinforcing polymyxin’s role as a last-line agent for these difficult-to-treat pathogens (22–24). Observational clinical data also supports the use of meropenem containing combinations, which have been associated with lower mortality rates (25). Additionally, a small prospective study found that intravenous fosfomycin-based combination therapy reduced all-cause hospital mortality in critically ill patients with CRKP infections (26).

Meropenem and fosfomycin were selected for their different but complementary mechanisms of action: meropenem inhibits peptidoglycan synthesis by binding to penicillin-binding proteins, while fosfomycin prevents the transpeptidation of peptidoglycan, an earlier step in peptidoglycan biosynthesis. Both drugs are hydrophilic, and mutations in outer membrane porins can hinder their ability to penetrate and achieve adequate exposure at the site of infection. Polymyxin B, through its detergent-like disruption of the outer membrane, enhances the intracellular accumulation of meropenem and fosfomycin (22, 27). Recent Infectious Diseases Society of America (IDSA) guidance does not recommend polymyxin-based combination regimens for CRE infections due to the increased toxicity associated with this narrow therapeutic index drug (2). Despite these concerns over nephrotoxicity, various clinical trials like OVERCOME and AIDA have demonstrated reduced mortality in CRE with polymyxin-based combination therapies (14, 15).

To assess the synergistic potential of polymyxin B with meropenem and/or fosfomycin, we selected six different isolates with varying phenotypic and genotypic resistance mechanisms. These included strains harboring carbapenemases like *bla*_KPC-2_ or *bla*_NDM-4_*,* with or without porin mutations that confer resistance to meropenem. Fosfomycin uptake is facilitated by *ompF* in *E. coli,* a homolog of *ompK35* in *K. pneumoniae* (28)*;* five of the six isolates had *ompK35* mutations, potentially limiting fosfomycin penetration. Two isolates also carried *MgrB* mutations, one of which (BRKP28) exhibited extreme phenotypic resistance to polymyxin B (>128 mg/L) (Table S2).

Information about phenotypic and genotypic resistance mechanisms provided significant insights into the mechanistic synergy in MBM across the six isolates. Based on the presence of porin mutations and polymyxin B susceptibility, mechanistic synergy was applicable to only three isolates. Our model estimated 83%–88% mechanistic synergy for polymyxin B with meropenem and 81%–98% with fosfomycin. While polymyxin B-meropenem combinations demonstrated >80% synergy, they did not produce meaningful reduction in AUC_CFU. Importantly, the synergistic effect was evident as an initial ~2 log_10_ CFU/mL reduction within the first 4 h for most isolates. This early bactericidal activity could be clinically relevant, particularly when considered in the context of host immune response. However, in the time-kill approach, this initial strong activity is followed by complete regrowth. Therefore, further *in vivo* studies evaluating this combination may give some insights into treatment optimization with this combination. In contrast, the 81%–98% synergy of polymyxin B-fosfomycin combinations led to substantial AUC_CFU reductions for four isolates.

Findings from the present study collectively guided the development of a decision-support workflow (Fig. 5), to inform antibiotic selection based on carbapenemase production and polymyxin B susceptibility for clinical use or further dynamic *in vitro* evaluation (e.g., hollow fiber infection models). Alternative dosing strategies, such as front-loading polymyxin B or site-specific administration (e.g., inhaled polymyxin B for lung infections), may further enhance therapeutic efficacy (23).

The early bactericidal activity observed with the polymyxin B–meropenem combination in the present study may have clinical relevance; however, the subsequent regrowth underscores the challenge of managing CRKP infections with this regimen in the USA, where IV fosfomycin is not yet approved. Notably, a clinical trial of IV fosfomycin (ZTI-01) for complicated urinary tract infections and pyelonephritis demonstrated favorable efficacy and safety (29). Fosfomycin monotherapy against BRKP28 isolate showed substantial initial decrease of 2-3 log_10_ CFU/mL within the first 8 h, which could be clinically meaningful when considering the host immune response. However, in the time-kill assay, where immune factors are absent, this early bactericidal effect was followed by complete bacterial regrowth. The addition of polymyxin B did not alter the regrowth pattern, suggesting that the current antibiotic combinations lack sustained efficacy against this strain. Hence, further investigation into β-lactam/β-lactamase inhibitor combinations administered 8 h after initial fosfomycin-based treatment in *in vivo* models may offer promising therapeutic insights for treating BRKP28 effectively.

In the present study, polymyxin B-based combinations were effective against all isolates except BRKP28. This is likely due to uncharacterized resistance mechanisms, possibly involving positively charged lipopolysaccharide (LPS) that interferes with polymyxin B’s interaction with LPS. Resistance to polymyxin B is increasingly reported and is often driven by mutations in two-component systems (TCS) such as *PhoP/PhoQ* and *PmrA/PmrB*, which regulate the expression of genes involved in LPS modification. These mutations result in the addition of cationic groups such as 4-amino-4-deoxy-L-arabinose (L-Ara4N) or phosphoethanolamine (PEtn) to lipid A, reducing the net negative charge of the bacterial surface and weakening electrostatic interactions with polymyxins (30). Additionally, loss of *mgrB*, a negative regulator of the *PhoQ/PhoP* pathway, further enhances L-Ara4N modification of lipid A. Such alterations of polymyxin B’s target in LPS contribute to increased resistance to polymyxins (31).

Despite these resistance mechanisms, combination therapies remain effective. For example, a recent study found that the triple combination therapy with polymyxin B, meropenem and fosfomycin significantly suppressed the selective amplification of polymyxin B resistant subpopulations in *Acinetobacter baumannii* infections (32). Consistent with this, our results demonstrate that polymyxin B-based combinations are effective against polymyxin-resistant isolate like BRKP67, suggesting that the polymyxin B MIC (i.e., phenotypic susceptibility) alone may not be predictive of the pharmacodynamic response. Supporting this, MacNair et al. reported that *Enterobacteriaceae* strains harboring *mcr-1* did not impede colistin’s ability to disrupt the outer membrane, indicating that acquired resistance to polymyxins does not necessarily hinder its synergistic potential in combination (33). Similarly, Sharma et al. observed extensive morphological changes in all isolates except BRKP28 following polymyxin B exposure (27).

Concerns about nephrotoxicity with higher polymyxin B exposure remain. Tailored polymyxin B-based combination therapy is essential to ensure efficacy while minimizing the risk for nephrotoxicity (23). A retrospective study in adult patients found a 50% probability of polymyxin B-associated acute kidney injury when trough concentrations (total drug) ≥3.13 mg/L (34). Our simulations showed that a low-dose polymyxin B regimen (1 mg/kg every 12 h) maintained trough concentrations below the nephrotoxic threshold while still achieving synergistic effects. Given fosfomycin’s favorable safety profile, its maximum recommended dose (8 g every 8 h) should be used to maximize synergistic effects. In cases requiring triple combination therapy, adding low dose meropenem (1 g every 8 h) or the recommended meropenem regimen (2 g every 8 h) is necessary to achieve bacterial reduction. Both meropenem regimens maintained trough concentrations (total drug) below the nephrotoxic (44.5 mg/L) and neurotoxic (64.5 mg/L) thresholds (35) (Fig. S1).

Dosing strategies recommended in this study were derived from our MBM, which applied a consistent structural growth model across all six isolates, incorporating distinct subpopulation dynamics and drug-specific killing effects. While our findings align with prior systematic reviews addressing therapeutic approaches for multidrug-resistant Gram-negative bacteria (36, 37) and our modeling approach mirrors previous MBM applications, its generalizability is limited by the resistance mechanisms expressed by each strain and its polymyxin B susceptibility. In contrast, a prior MBM model developed using six *E. coli* isogenic strains, successfully generalized to predict ciprofloxacin efficacy in additional strains not used in model development. The study employed a similar structural framework across strains and allowed potency parameters (EC50) to vary, reflecting differences in susceptibility, and accurately predicted outcomes under varied inoculum conditions (38). Similarly, another study integrated clinical PK/PD data from the AIDA trial into MBM, incorporating isolate-specific drug effect parameters, and found a correlation between individualized predictions of bacterial burden and clinical trial outcomes (39).

Future studies should expand evaluation of polymyxin B-based combinations and novel β-lactamase inhibitors across a broader panel of CRKP isolates with diverse genotypes. Furthermore, the mechanism-based model can be improved by integrating multi-omics data to enhance understanding of how specific resistance mechanisms influence drug pharmacodynamic activity in monotherapy and combination regimens to improve the applicability of a MBM approach to select therapies effective against more strains with different resistance mechanism.

## MATERIALS AND METHODS

### Antimicrobials and media

Mueller-Hinton broth (MHB; Becton Dickinson, Franklin Lakes, NJ) supplemented with calcium and magnesium (CAMHB; 25.0 mg/L Ca^2+^, 12.5 mg/L Mg^2+^) and Mueller-Hinton II agar (MHA; Becton, Dickinson, Franklin Lakes, NJ) were used for susceptibility testing and all *in vitro* experiments. CAMHB was further supplemented with 25 mg/L glucose-6-phosphate (G-6-P) (lot number 343234, Acros Organics). The inclusion of G-6-P enhances the *in vitro* activity of fosfomycin, aligning it more closely with *in vivo* efficacy. This is because G-6-P compensates for reduced bacterial uptake of fosfomycin caused by the presence of glucose and phosphate in Mueller-Hinton agar (40). Stock solutions of polymyxin B (lot number WXBB5309V; Sigma Aldrich, St. Louis, MO), meropenem (lot number LC24337; AK Scientific, Union City, CA) and fosfomycin (lot number: K001, Nabriva Therapeutics US, Inc., Fort Washington, PA) were freshly prepared in sterile water and saline prior to each experiment. All drug solutions were filter sterilized with a 0.22 µm filter (Fisher Scientific, Pittsburgh, PA).

### Bacterial isolates and antibiotic susceptibility testing

Four clinical CRKP isolates used in this study (BRKP28, BRKP61, BRKP67, BRKP76) were obtained from individual patients at Instituto Dante Pazzanese de Cardiologia (Sao Paulo, Brazil), while two additional isolates (KP0016-1 and KP0052-1) were obtained from Siriraj Hospital (Bangkok, Thailand) (27). Polymyxin B and meropenem MICs were determined in triplicate using the broth microdilution method, whereas fosfomycin susceptibility was assessed using the agar dilution method, both following CLSI guidelines (41). MIC interpretations for polymyxin B and meropenem were based on CLSI breakpoints for *K. pneumoniae*. Since CLSI does not provide breakpoints for fosfomycin against *K. pneumoniae*, EUCAST for intravenous fosfomycin against *E. coli* were used as a reference (42).

### Genomic characterization

Polymerase chain reaction (PCR) was performed using previously described primer sets for β-lactamase Ambler classes A (*bla*_GES_ and *bla*_KPC_), B (*bla*_NDM-1_, *bla*_VIM_, and *bla*_IMP_), and D (*bla*_OXA-48_ and *bla*_OXA-40_) (43). Primers used in amplification experiments to identify *mgrB* (31) and *ompK* genes (44) (*ompK35*, *ompK36,* and *ompK37*) are listed in Table S1. Isolates were also characterized for the virulence genes and the primers used for the virulence genes are mentioned in Table S1. Genomic DNA was extracted from bacterial isolates using the Purelink Genomic DNA Mini kit (Invitrogen, Carlsbad, CA). PCRs were performed using Q5 Hi-Fidelity Taq DNA Polymerase (New England Biolabs, Ipswich, MA). Reactions were carried out in an Eppendorf Mastercycler (Eppendorf, Hamburg, Germany), and PCR products were analyzed by direct sequencing (GENEWIZ, Research Triangle Park, NC). Nucleotide and deduced protein sequences were analyzed using BLAST (http://blast.ncbi.nlm.nih.gov/).

### Static concentration time-kill assays

SCTK assays were conducted over a 24 h period to evaluate the rate and extent of killing by monotherapy, double and triple combinations using polymyxin B, meropenem, and fosfomycin. PK/PD analysis was performed using six isolates at a starting inoculum of ~10^6^ CFU/mL (CFU_0_), as previously described (45). Antibiotic concentrations selected included both clinically achievable (polymyxin B: 0.5, 1, 2, 4, 8 mg/L; meropenem: 10, 20, 40 mg/L; Fosfomycin: 75, 150, 300, 500 mg/L) and supra-therapeutic (polymyxin B: 16 and 64 mg/L; meropenem: 60 and 120 mg/L) free-drug concentrations (i.e., unbound plasma concentrations) to characterize the concentration-response relationship against each isolate. Clinically relevant concentrations were selected based on maximum plasma concentrations (Cmax) reported in previously published population PK studies (17, 46, 47). Serial samples were obtained at 0, 1, 2, 4, 6, 8, and 24 h for bacterial quantification, with a lower limit of quantification (LLOQ) of 2 log_10_ CFU/mL.

### Pharmacodynamic analysis

The pharmacodynamic effect was quantified as the change in log_10_ CFU/mL at 24 h (CFU*_24_*) compared to baseline (CFU_0_) (i.e., 24 h log_10_ CFU/mL reduction). Bactericidal activity was defined as a ≥ 3 log_10_ reduction at 24 h compared to the initial inoculum. To further assess bacterial killing in the polymyxin B-based combination therapy, AUC___CFU from 0 to 24 hours was calculated for both the double and triple drug combinations at static concentrations.

### Mechanism based PK/PD model development

Mechanism-based PK/PD model was developed using the SCTK time course data describing the change in bacterial burden over time in response to antibiotic treatment against the six bacterial isolates. A life cycle model was used to describe the bacterial growth and replication for each bacterial isolate (48). The model included the transition of bacterial cells from vegetative state, preparing for replication (state 1), to the replication state, immediately prior to replication step (state 2).

A mixture model with pre-existing subpopulations with differing susceptibilities to polymyxin B, meropenem, and fosfomycin was considered to account for heterogeneity in the bacterial inoculum. Models with two, three, and four bacterial subpopulations were explored (49). The final model included two subpopulations with differing susceptibilities to polymyxin, meropenem, and fosfomycin for each isolate (equation 1).


(1) $$CFU_{tot}=CFU_{IRR,1}+CFU_{IRR,2}+CFU_{RII,1}+CFU_{RII,2}$$

where *CFU_tot_* is the total viable bacterial concentration, *CFU_IRR_* is the polymyxin B-intermediate, meropenem-resistant and fosfomycin-resistant subpopulation. *CFU_RII_* is the polymyxin B-resistant, meropenem-intermediate and fosfomycin-intermediate subpopulation. Each of the bacterial subpopulations, *CFU_IRR,1_* and *CFU_IRR,2_,* is in vegetative (state 1) and replicative state (state 2) respectively. The differential equation for the two states describing the bacteria in state 1 and 2 for subpopulation *CFU_IRR_* with killing by polymyxin B (PMB), meropenem (MEM), and fosfomycin (FOF) is shown in equations 2 and 3.


(2) $$\begin{array}{rl} & \frac{d\left(CFU_{IRR,1}\right)}{dt}=REP\cdot k_{21}\cdot CFU_{IRR,2}-k_{12,IRR}\cdot CFU_{IRR,1}-\left(Kill_{PMB,I}+Kill_{MEM,R}+Kill_{FOF,R}\right)\cdot \\ & CFU_{IRR,1},\left(IC_{CFU_{IRR,1}}=CFU_{0}\cdot MF_{IRR}\right) \end{array}$$

(3) $$\begin{array}{rl} & \frac{d\left(CFU_{IRR,2}\right)}{dt}=-k_{21}\cdot CFU_{IRR,2}+k_{12,IRR}\cdot CFU_{IRR,1}-\left(Kill_{PMB,I}+{\mathrm{Kill}}_{MEM,R}+{\mathrm{Kill}}_{FOF,R}\right)\cdot \\ & CFU_{IRR,2},\left(IC_{CFU_{IRR,2}}=0\right) \end{array}$$

where REP is the replication factor defined as $2\cdot \left(1-\frac{CFU_{tot}}{CFU_{MAX}+CFU_{tot}}\right)$, *CFU_MAX_* is the maximum bacterial population size and 2 represents the doubling of bacteria during replication. The inverse of the mean replication time from state 2 to state 1, *k_21_* was fixed to 50 h^−1^ (49, 50). *k_12, IRR_*, the inverse of mean replication time from state 1 to state 2 was estimated for each subpopulation. *Kill_PMB_,_I_*, *Kill_MEM_,_R_,* and *Kill_FOF_,_R_* are the killing rate by polymyxin B, meropenem, and fosfomycin, respectively for *CFU_IRR_* subpopulation. The killing activity of each drug is described by the Hill equation as shown in equation 4.


(4) $$Kill_{drug}=\frac{K_{MAX,drug\;}\cdot \;drug^{h}}{{\left(KC_{50,drug}\right)}^{h}+drug^{h}}$$

where drug can be concentration of polymyxin B, meropenem, or fosfomycin; *K_MAX,drug_* is the maximum killing rate constant of drug; *KC_50,drug_* is the drug concentration causing 50% of *K_MAX,drug_*; h is the Hill coefficient. We considered subpopulation synergy (i.e., polymyxin B killing bacteria resistant to meropenem/fosfomycin and vice versa) in this model. We estimated the total initial inoculum (log_10_CFU_0_) and the log transformed mutation frequency (MF) for the subpopulations. Initial conditions were implemented as described previously (49).

The interaction between polymyxin B and meropenem and/or fosfomycin was modeled using a Hill function to describe the mechanistic synergy based on polymyxin B’s effect on the outer membrane of the Gram-negative bacteria. This synergy increases the target site concentration of meropenem and fosfomycin, thereby enhancing the sensitivity of the intermediate and resistant subpopulations to meropenem and fosfomycin (*KC_50,MEM,I_*, *KC_50,FOF,I_, KC_50,MEM,R_*, *KC_50,FOF,R_*). Equation 5 describes the mechanistic synergy by polymyxin B, and equations 6 and 7 describes the impact of mechanistic synergy on meropenem intermediate and fosfomycin resistant subpopulation, respectively.


(5) $$Mechanistic\_synergy=1-\left(\frac{I_{MAX}\cdot (C_{PMB}{)}^{h}}{(C_{PMB}{)}^{h}+(IC_{50}{)}^{h}}\right)$$

(6) $$Kill_{MEM,I}=\frac{K_{MAX,MEM}\cdot MEM^{h}}{{\left(KC_{50,MEM,I}\cdot Mechanistic\_synergy\right)}^{h}+MEM^{h}}$$

(7) $$Kill_{FOF,R}=\frac{K_{MAX,FOF\;}\cdot \;FOF^{h}}{{\left(KC_{50,FOF,R}\cdot Mechanistic\_synergy\right)}^{h}+FOF^{h}}$$

Where *I_MAX_* is the maximum fractional decrease in *KC_50,MEM_,_I_* and *KC_50,FOF,R_* by polymyxin B causing disruption of the bacterial outer membrane, *IC_50_* is the polymyxin B concentration causing 50% of *I_MAX_*, *h* is the hill coefficient.

The residual unexplained variability for log_10_ transformed bacterial load data was explained by additive error model. Observations below the LLOQ were fit using the Beal M3 (51). Parameter estimation was performed using the importance sampling algorithm (pmethod = 4) in S-ADAPT (version 1.57) facilitated by SADAPT-TRAN (52). Models were evaluated based on model fits, diagnostic plots, precision of parameter estimates, and objective function value (OFV).

### Monte Carlo simulations to predict drug response in clinical practice

Monte Carlo simulations were performed for 1,000 adult patients using mrgsolve R package. Previously published population PK models for polymyxin B (17), meropenem (46), and fosfomycin (47) in critically ill patients were used to simulate the unbound plasma concentrations taking inter individual variability into consideration (Fig. S1). The unbound plasma concentrations were determined using published fraction unbound (*fu*) for polymyxin B = 0.42 and meropenem = 0.98, while plasma protein binding for fosfomycin was negligible. These simulated unbound drug concentrations were linked to the developed MBM to predict impact on bacterial killing in critically ill patients. Simulations were performed using demographics within the reported range in the population PK models for polymyxin B, meropenem, and fosfomycin [bodyweight of 70 kg, serum albumin of 2.8 g/dl, and creatinine clearance of 90 mL/min].

The efficacy of a lower polymyxin B dosing regimen (1 mg/kg every 12 h as 1 h infusion) was evaluated in both the double and triple combination regimens. In cases where the lower dosing regimen did not result in a significant reduction in AUC_CFU, simulations were performed using the recommended weight-based polymyxin B regimen (2.5 mg/kg loading dose [LD] followed by 1.5 mg/kg every 12 h maintenance dose [MD] as 1 h infusion) and a fixed regimen (150 mg LD and 75 mg every 12 h MD as 1 h infusion). Additionally, meropenem (1 g or 2 g every 8 h as 3 h infusion) and fosfomycin (4 g or 8 g every 8 h as 3 h infusion) regimens were simulated. The pharmacodynamic activity of double and triple combination therapy was evaluated as a percentage reduction in AUC_CFU with the treatment of interest compared to no treatment.

### Conclusion

While the importance of appropriate antimicrobial therapy is well recognized, high-quality evidence to guide antibiotic selection for extensively drug resistant CRE isolates is limited. Addressing this gap, a key contribution of this work is the development of a mechanism-based PK/PD model informed by resistance mechanisms. Model-based simulations, leveraging clinical drug exposure from published population PK model, enabled prediction of pharmacodynamic responses in critically ill patients. Notably, our findings indicate that a low-dose polymyxin B regimen (1 mg/kg q12h) can produce synergistic effects when combined with appropriate companion antibiotic(s) while minimizing nephrotoxicity. Such combinations can be effective in managing infections caused by both carbapenamase producing and non-producing CRKP, strains including those resistant to polymyxin B.

Our results also suggest that polymyxin B shows greater synergy with fosfomycin than with meropenem. Triple combination therapy involving polymyxin B, meropenem, and fosfomycin may be particularly beneficial for non-carbapenamase producing isolates resistant to newer antibiotics. Understanding the underlying resistance mechanisms is essential for designing treatment regimens that combine both older and newer antibiotics to achieve adequate exposure at the infection site.

Future research should focus on integrating genotype-phenotype associations with quantitative pharmacodynamic data, especially to evaluate drug activity in the presence or absence of specific resistance genes. Given the limited clinical data, a systematic and rational approach to generating robust nonclinical PK/PD evidence is critical for preserving the effectiveness of our current antibiotic arsenal.

## SUPPLEMENTAL MATERIAL

Supplemental figures and tables aac.00782-25-s0001.docx Figures S1-S5 and Tables S1 and S2.

ASM does not own the copyrights to Supplemental Material that may be linked to, or accessed through, an article. The authors have granted ASM a non-exclusive, world-wide license to publish the Supplemental Material files. Please contact the corresponding author directly for reuse.
