# Supplementary material for: A mechanism-based pharmacokinetic/pharmacodynamic analysis of polymyxin B-based combination therapy against carbapenem-resistant Klebsiella pneumoniae isolates with diverse phenotypic and genotypic resistance mechanisms
Source: Antimicrob Agents Chemother. 2025 Dec 19;70(2):e00782-25. doi: 10.1128/aac.00782-25 (PMC12888851; doi:10.1128/aac.00782-25)
Supplement: Supplemental figures and tables — Figures S1-S5 and Tables S1 and S2. [file aac.00782-25-s0001.docx]

**Supplemental Material**

**A Mechanism Based Pharmacokinetic/Pharmacodynamic Analysis of Polymyxin B-Based Combination Therapy Against Carbapenem-Resistant *Klebsiella pneumoniae* Isolates with Diverse Phenotypic and Genotypic Resistance Mechanisms**

Ramya Mahadevan^1^*, Estefany Garcia^2^*, Rajnikant Sharma^1^, Hongqiang Qiu^2,3,4^, Ahmed Elsheikh^2^, Robert Parambi^2^, Cely Saad Abboud^5^, Fernando Pasteran^7^, Maria Soledad Ramirez^8^, Keith S. Kaye^6^, Robert A. Bonomo ^9, 10, 11, 12,13^, Gauri G. Rao^1,2^

**Corresponding author:** Dr. Gauri G. Rao, Titus Family Department of Clinical Practice, USC Alfred E. Mann School of Pharmacy and Pharmaceutical Sciences, University of Southern California, Los Angeles, California, USA E-mail: gaurirao@usc.edu

**Short title:** Polymyxin B combination therapy effective against CRKP

**Table S1:** PCR primers used for amplification in this study.

| **Gene(s)** | **Sense primer** | **Reverse primer** |
| --- | --- | --- |
| *bla*_KPC_ | TCGCTAAACTCGAACAGG | TTACTGCCCGTTGACGCCCAATCC |
| *bla*_NDM-1_ | ATGGAATTGCCCAATATT | TCAGCGCAGCTTGTCGGC |
| *bla*_GES_ | CTATTACTGGCAGGGATCG | CCTCTCAATGGTGTGGGT |
| *bla*_OXA-48_ | TGTTTTTGGTGGCATCGAT | GTAAMRATGCTTGGTTCGC |
| *bla*_IMP_ | GAGTGGCTTAATTCTCRATC | AACTAYCCAATAYRTAAC |
| *bla*_VIM_ | GTTTGGTCGCATATCGCAAC | AATGCGCAGCACCAGGATAG |
| *bla*_OXA-40_ | CACCTATGGTAATGCTCTTGC | GTGGAGTAACACCCATTCC |
| *mgrB* | TTAAGAAGGCCGTGCTATCC | AAGGCGTTCATTCTACCACC |
| *ompk35* | CAGACACCAAACTCTCATCAATGG | AGAATTGGTAAACGATACCCACG |
| *ompk36* | CAGCACAATGAATATAGCCGAC | GCTGTTGTCGTCCAGCAGGTTG |
| *ompk37* | CATTCCGCAGAATGAGACGGCAAC | CGACGATGTTATCGGTAGAGATAC |
| *mrkD* | TCAGATGCGAAAGTTGTTGC | AGGTTCACATCCCTGTTGTG |
| *fimH* | ATTGCGACACTACGGTAGCC | ATGATGTTGGGGTCGTCATT |
| *wabG* | CGGACTGGCAGATCCATATC | ACCATCGGCCATTTGATAGA -3' |
| *kfuBC* | GAAGTG ACGCTGTTTCTGGC | TTTCGTGTGGCCAGTGACTC |
| *cf29a* | GACTCTGATTGCACTGGCTGTG | GTTATA AGTTACTGCCACGTTC |
| *uge* | GCTGACTTAAGAGAACGTTATG | GATCATGGCGCTACCT(C/T) A |
| *rmpA* | ACTGGGCTACCTCTGCTTCA | CTTGCATGAGCCATCTTTCA |
| *allS* | CCGTTAGGCAATCCAGAC | TCTGATTTA (A/T)CCCACATT |
| *magA* | GGTGCTCTTTACATCATTGC | GCAATGGCCATTTGCGTTAG |

**Table S2:** Genomic characterisation of six multidrug-resistant *Klebsiella pneumoniae* strains isolated from patients.

| Strains | BRKP61 | BRKP67 | BRKP76 | BRKP28 | KP0016-1 | KP0052-1 |
| --- | --- | --- | --- | --- | --- | --- |
| *KPC-2* | Comparable in size with that in wild-type | Comparable in size with that in wild-type | Comparable in size with that in wild-type | Comparable in size with that in wild-type | Not amplified | Comparable in size with that in wild-type |
| *GES* | Not amplified | Not amplified | Not amplified | Not amplified | Not amplified | Not amplified |
| *NDM* | Not amplified | Not amplified | Not amplified | Not amplified | Not amplified | NDM-4 |
| *VIM* | Not amplified | Not amplified | Not amplified | Not amplified | Not amplified | Not amplified |
| *IMP* | Not amplified | Not amplified | Not amplified | Not amplified | Not amplified | Not amplified |
| *OXA40* | Not amplified | Not amplified | Not amplified | Not amplified | Not amplified | Not amplified |
| *OXA48* | Not amplified | Not amplified | Not amplified | Not amplified | Not amplified | Not amplified |
| *mgrB* | Comparable in size with that in wild-type | Insertion of ISKpn13 (1148 bp), an IS5-like element | Comparable in size with that in wild-type | Premature stop codon | Comparable in size with that in wild-type | Comparable in size with that in wild-type |
| *Ompk-35* | Not amplified | Terminated at 144 AA | Terminated at 144 AA | Terminated at 144 AA | Terminated at 91 AA | Comparable in size with that in wild-type |
| *Ompk-36* | Stop codon at 2nd AA | Few AA substitutions | Few AA substitutions | Not amplified | Not amplified | Comparable in size with that in wild-type |
| *Ompk-37* | Comparable in size with that in wild-type | Comparable in size with that in wild-type | Few AA substitutions | Comparable in size with that in wild-type | Not amplified | Comparable in size with that in wild-type |
| *magA* | Not amplified | Not amplified | Not amplified | Not amplified | Not amplified | Not amplified |
| *allS* | Not amplified | Not amplified | Not amplified | Not amplified | Not amplified | Not amplified |
| *mrkD* | Not amplified | Not amplified | Not amplified | Not amplified | Not amplified | Not amplified |
| *fimH* | Present | Present | Present | Present | Not amplified | Present |
| *rmpA* | Not amplified | Not amplified | Not amplified | Not amplified | Not amplified | Not amplified |
| *uge* | Not amplified | Not amplified | Not amplified | Not amplified | Not amplified | Not amplified |
| *cf29a* | Not amplified | Not amplified | Not amplified | Not amplified | Not amplified | Not amplified |
| *kfuBC* | Present | Present | Present | Present | Not amplified | Present |
| *wabG* | Present | Present | Present | Present | Present | Present |

**
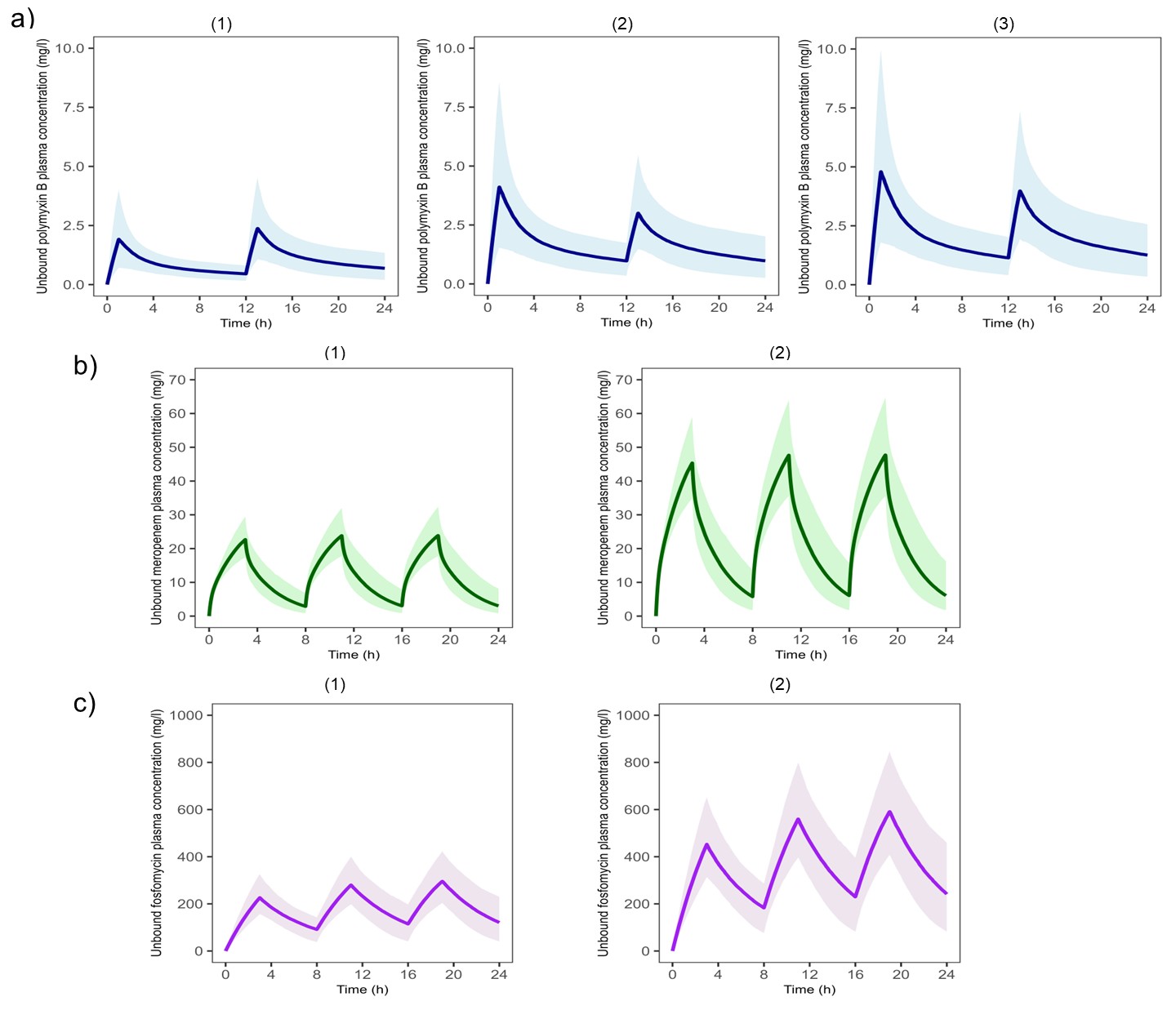
**

**Figure S1:** Simulated unbound plasma concentration–time profiles. a) Polymyxin B: (1) 1 mg/kg q12h; (2) fixed dosing with a 150 mg loading dose (LD) and 75 mg maintenance dose (MD) q12h; (3) weight-based dosing with 2.5 mg/kg LD and 1.5 mg/kg MD q12h. Polymyxin B was administered as a 1h inf, with the MD initiated 12 h after completion of the LD. b) Meropenem: (1) 1 g q8h; (2) 2 g q8h. c) Fosfomycin: (1) 4 g q8h; (2) 8 g q8h. Both meropenem and fosfomycin were administered as 3-h infusions.


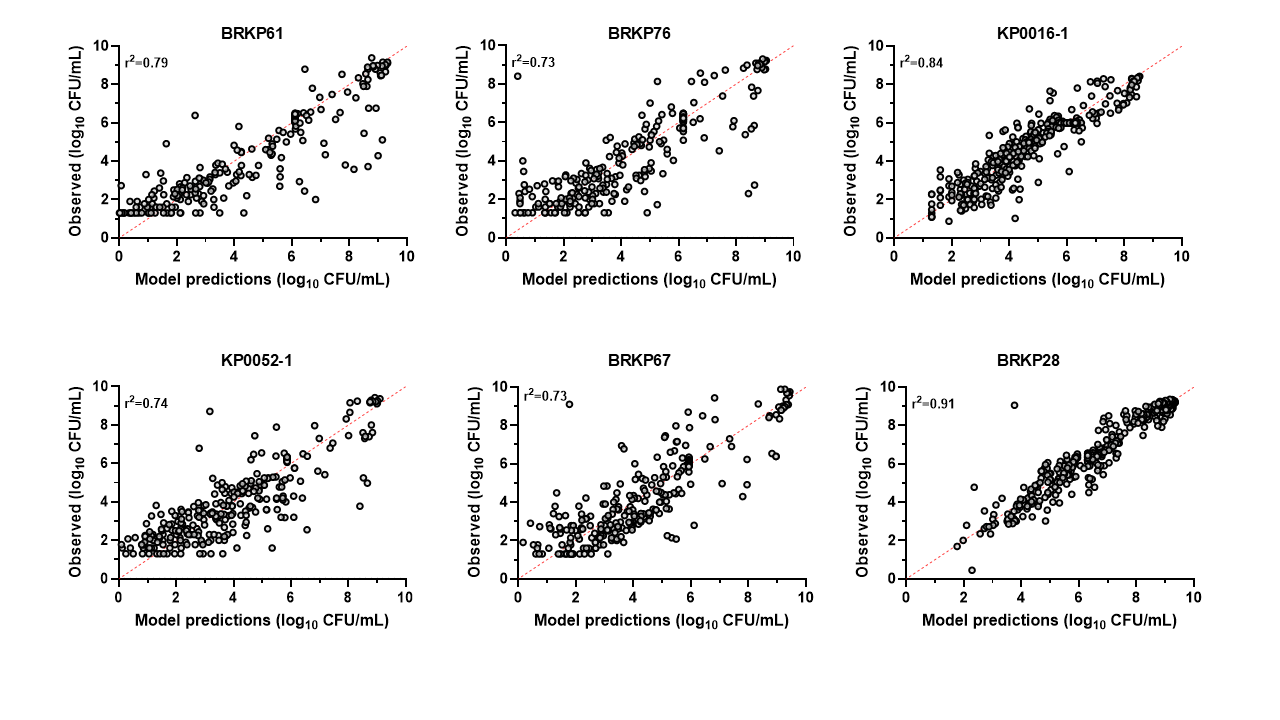


**Figure S2:** Observed data vs model predictions for six isolates.


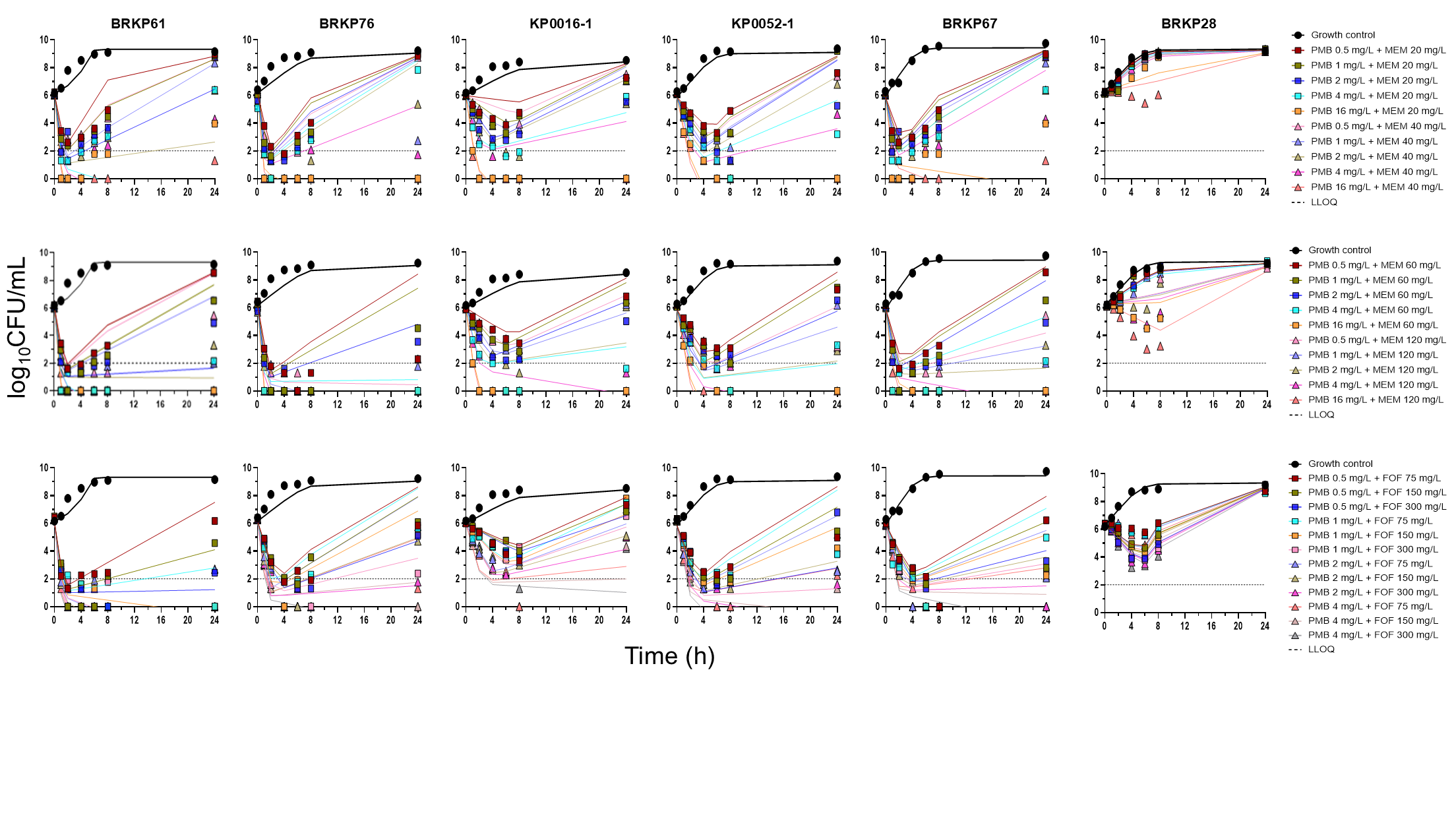
**Figure S3:** Model predictions for double combination therapy across six isolates. The solid lines represent model predictions, and the symbols denote observed data. The LLOQ is 2 log_10_ CFU/mL.


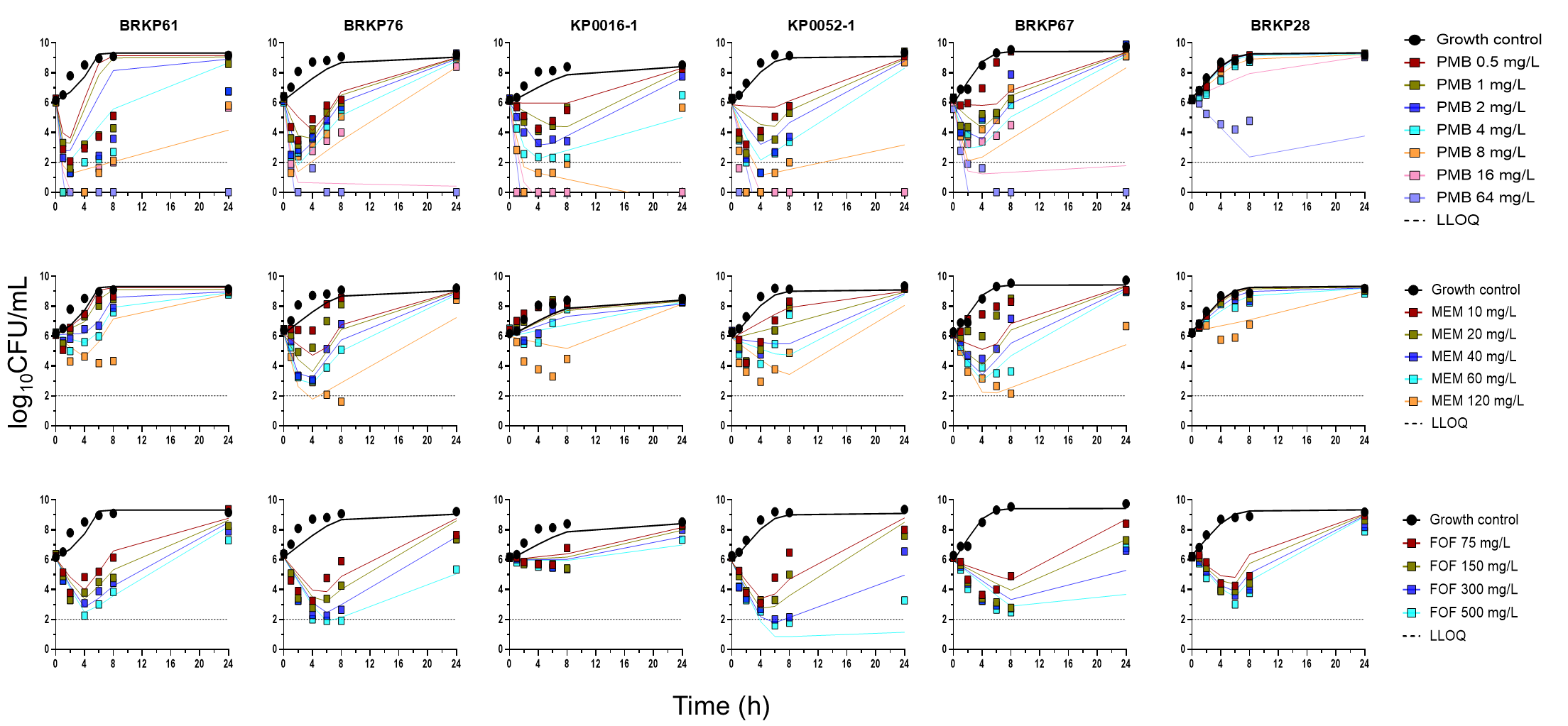


**Figure S4:** Model predictions for monotherapy across six isolates. The solid lines represent model predictions, and the symbols denote observed data. The LLOQ is 2 log_10_ CFU/mL.


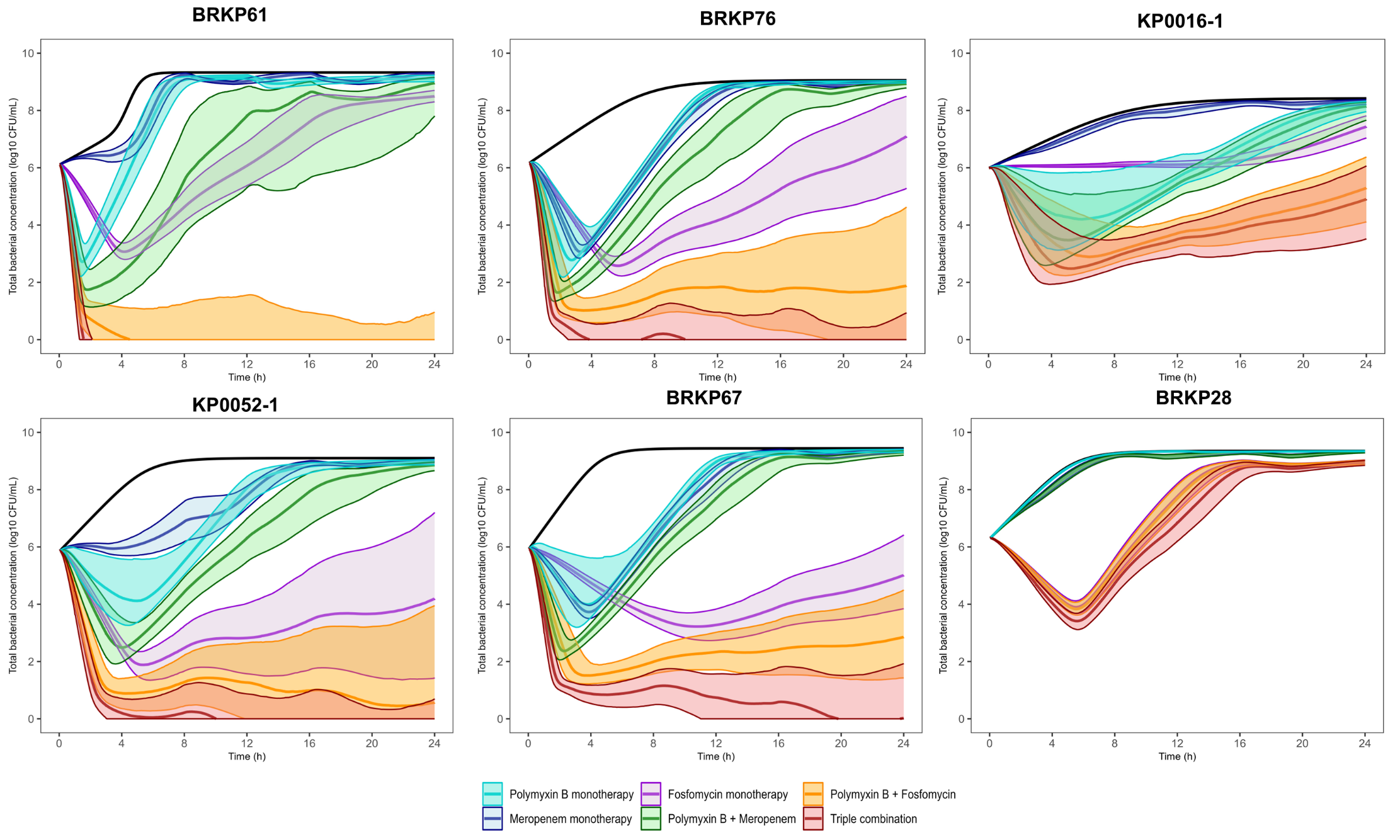


**Figure S5:** Model-predicted bacterial count profiles following treatment with monotherapy, double, and triple combination regimens for six isolates. Polymyxin B was given at 1 mg/kg q12h (1h inf), meropenem at 2 g q8h (3h inf), and fosfomycin at 8 g q8h (3h inf). The solid lines represent the median predictions, and the shaded areas indicate the 90% prediction intervals based on 1000 simulated patients.
